# Exercise Alleviates Osteoporosis in Rats with Mild Chronic Kidney Disease by Decreasing Sclerostin Production

**DOI:** 10.3390/ijms20082044

**Published:** 2019-04-25

**Authors:** Hung-Wei Liao, Tsang-Hai Huang, Yi-Han Chang, Hung-Hsiang Liou, Yu-Hsien Chou, Yuh-Mou Sue, Peir-Haur Hung, Yu-Tzu Chang, Pei-Chuan Ho, Kuen-Jer Tsai

**Affiliations:** 1 Chinru Clinic, Taipei 116, Taiwan; lhw898@gmail.com; 2 Institute of Physical Education, Health and Leisure Studies, National Cheng Kung University, Tainan 704, Taiwan; tsanghai@mail.ncku.edu.tw (T.-H.H.); powerkobe2007@hotmail.com (Y.-H.C.); 3 Institute of Clinical Medicine, College of Medicine, National Cheng Kung University, Tainan 704, Taiwan; jdmb1121@gmail.com (Y.-H.C.); peggy821124@gmail.com (P.-C.H.); 4 Division of Nephrology, Department of Medicine, Hsin-Jen Hospital, New Taipei City 242, Taiwan; hh258527@ms23.hinet.net; 5 Division of Nephrology, Department of Internal Medicine, School of Medicine, College of Medicine and Wan Fang Hospital, Taipei Medical University, Taipei 116, Taiwan; sueym@tmu.edu.tw; 6 Department of Internal Medicine, Ditmanson Medical Foundation Chia-yi Christian Hospital, Chia-yi City 600; Taiwan; dtmedg3@yahoo.com.tw; 7 Division of Applied Life Science and Health, Chia-Nan University of Pharmacy and Science, Tainan 717, Taiwan; 8 Department of Internal Medicine, College of Medicine, National Cheng Kung University, Tainan 704, Taiwan; kangxiemperor@gmail.com; 9 Research center of Clinical Medicine, National Cheng Kung University Hospital, College of Medicine, National Cheng Kung University, Tainan 704, Taiwan

**Keywords:** exercise, chronic kidney disease, osteoporosis, sclerostin, chronic kidney disease–mineral bone disorder

## Abstract

Chronic kidney disease–mineral bone disorder (CKD–MBD), comprising mineral, hormonal, and bone metabolic imbalance, is a major CKD-related issue; it causes osteoporosis prevalence in CKD patients. Osteocyte-derived sclerostin inhibits the osteogenic Wnt/β-catenin signaling pathway; its levels rise when kidney function declines. Exercise modulates the physiological functions of osteocytes, potentially altering sclerostin production. It may aid bone and mineral electrolyte homeostasis in CKD. Mild CKD was induced in rats by partial nephrectomy. They were divided into: sham (no CKD), CKD, and CKD + exercise (8 weeks of treadmill running) groups. Micro-CT scanning demonstrated that the CKD + exercise-group rats had a higher bone mineral density (BMD) of the spine and femoral metaphysis and higher femoral trabecular bone volume than the CKD-group rats. Bone formation rates were not significantly different. The CKD + exercise-group rats had lower serum sclerostin (157.1 ± 21.1 vs 309 ± 38.1 pg/mL, *p* < 0.05) and CTX-1 (bone resorption marker) levels. Immunohistochemistry revealed higher tibial β-catenin concentrations in the CKD + exercise-group rats. Serum FGF-23, intact parathyroid hormone (iPTH), alkaline phosphatase (ALP), calcium, and phosphate levels showed no significant differences between these groups. Thus, exercise improves BMD and bone microstructure in mild CKD by inhibiting sclerostin production, but does not alter serum minerals.

## 1. Introduction

Chronic kidney disease has a high prevalence rate (>10%) in the general population [1]. In addition to being associated with a high risk of cardiovascular disease [2], chronic kidney disease–mineral bone disorder (CKD–MBD) is another major issue for CKD patients [3]. CKD–MBD represents complicated disturbances in parathyroid hormone, vitamin-D, fibroblast growth factor 23 (FGF-23), calcium, and phosphate levels, and bone turnover [4]. Disrupted homeostasis among these electrolytes, hormones, and bone metabolism not only leads to osteoporosis [5], which is mainly related to secondary hyperparathyroidism, but also increases vascular calcification [6,7]. Disruption of the bone and mineral electrolyte homeostasis and cardiovascular complications increase the mortality of CKD patients [8,9].

Currently, more abnormal protein secretions have been identified in CKD patients [10,11]. The levels of sclerostin, which is a negative regulator of bone growth [12], has been found to increase abnormally when the kidney function declines [10]. Sclerostin, which is secreted from osteocytes, inhibits the Wnt/β-catenin signaling pathway in bones and modulates bone mass [13]. Increased serum sclerostin levels are correlated with bone fracture [14]. Renal osteodystrophy in CKD is regarded as the disruption between bone formation by osteoblasts and bone resorption by osteoclasts [15]. The derivative treatments for CKD–MBD are correcting the electrolyte imbalance and inhibiting the excessive production of the parathyroid hormone [15]. In addition to anabolic osteoblasts and catabolic osteoclasts, osteocytes are also an important type of bone cells [16]. Osteocytes regulate osteoblastogenesis and osteoclastogenesis [17,18]. Since anti-sclerostin antibody treatment demonstrates bone mass increase [19], targeting ligands that are secreted by osteocytes probably affects the fate of consequent osteoporosis and mineral disturbance in CKD. Thus, osteocytes are the potential therapeutic targets for CKD–MBD [20]. 

The most commonly available drugs for osteoporosis in clinical practice are anti-catabolic drugs [21]. Moreover, the use of parathyroid hormone-based osteoanabolic drugs to treat CKD patients is controversial, since hyperparathyroidism in CKD is related to adverse cardiovascular outcomes [5]. Osteocytes, which act as mechanosensors and trigger bone modeling, are affected by exercise or mechanical stimulation [22,23]. Exercise is determined to improve bone mass and bone strength [24]. Active physical activity decreases the serum levels of osteocyte-derived sclerostin and contributes towards improving the serum bone turnover marker levels [25]. Whether exercise training in CKD patients alters sclerostin levels and further improves the CKD–MBD disorder is interesting. We designed a rat model of mild CKD, and subjected the animals to treadmill running; we then evaluated the effects of this exercise program on bone remodeling, serum biomarkers, and electrolyte homeostasis in rats with mild CKD. 

## 2. Results

### 2.1. Assessment of Serum and Urine Biochemistries

The severity of kidney failure induced in the CKD-group and the CKD + exercise-group rats was similar. Rats from both groups had mild CKD (blood urea nitrogen of around 40 mg/dL) [6], compared to those in the sham group, while the serum calcium and phosphate concentrations were not significantly different among the three experiment groups (Table 1). The CKD + exercise-group and the CKD-group rats showed a higher urinary fractional excretion of phosphate (FEP) than those in the sham group, which indicated that phosphate load was higher in the rats when kidney function declined. However, FEP showed no significant difference between the rats from the CKD and the CKD + exercise groups. Fractional excretion of calcium (FECa) was significantly high in the CKD-group rats, when compared with rats from the CKD + exercise group and the sham group (Table 1). Lower FECa in the CKD + exercise-group rats relative to the CKD-group rats indicated that exercise ameliorated calcium loss in the rats with kidney dysfunction.

### 2.2. Exercise Decreased the Levels of Circulating Sclerostin and Bone Resorption Markers in the Rats with Renal Dysfunction

**Figure 1:**
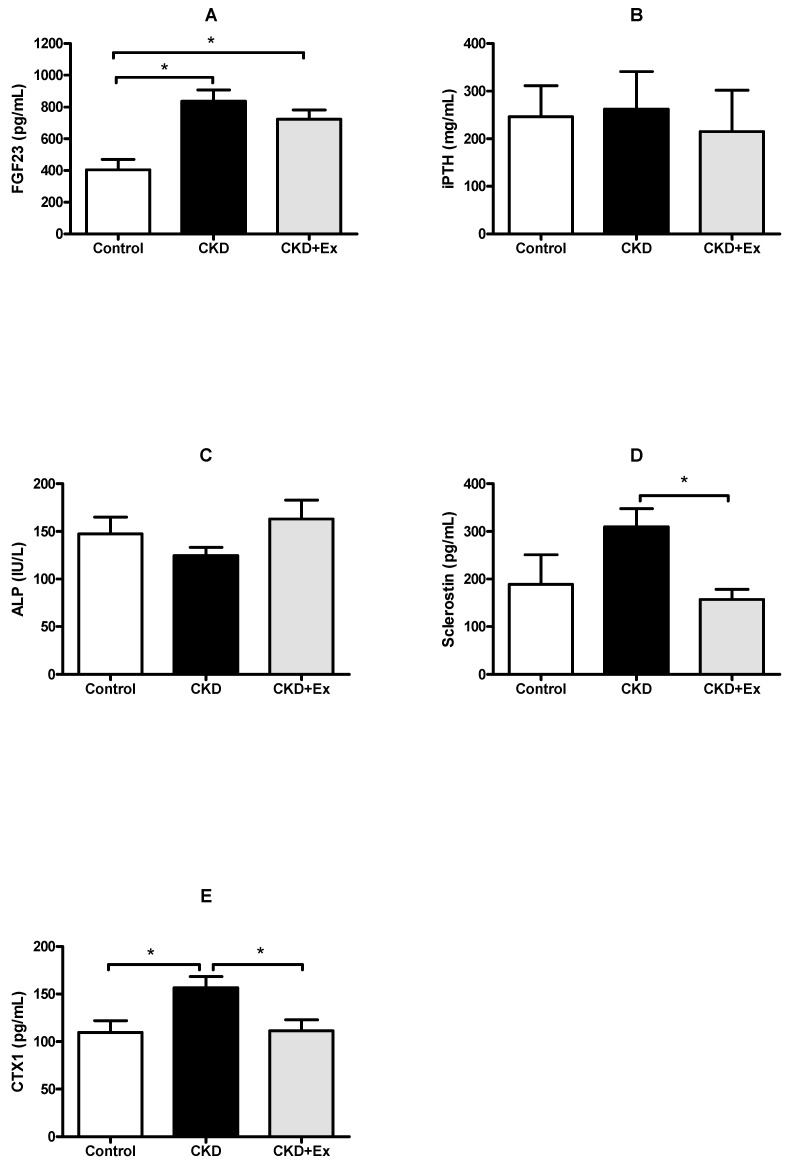
Serum biomarkers in the experimental groups. (**A**) Serum FGF-23 levels in rats from the CKD and the CKD + exercise (Ex) groups were higher than those from the sham group. (**B**,**C**) Serum intact parathyroid hormone (iPTH) and alkaline phosphatase (ALP) levels did not show significant differences between the three groups. (**D**) Serum sclerostin levels in rats from the CKD + exercise group were lower than those in rats from the CKD group. (**E**) Serum levels of CTX-1, a bone resorption marker, in rats from the CKD group were higher than those in rats from the CKD + exercise and the sham groups. *N* = 8, 13, and 9 in the control, CKD, and CKD + exercise groups, respectively. All data have been presented as mean ± SEM. * *p* < 0.05.

The serum FGF-23 levels were higher in the rats from the CKD + exercise group (723.9 ± 57.1 pg/mL) and the CKD group (836.1 ± 71.4 pg/mL) than those in the rats from the sham group (404.6 ± 64.8 pg/mL); this was because of the declined renal function in rats from the CKD and CKD + exercise groups (Figure 1A). The serum intact parathyroid hormone (iPTH) levels were not significantly different among the three experimental groups (Figure 1B). The serum iPTH levels did not increase significantly in the rats with kidney dysfunction; this was probably due to the mild CKD induced in the rats in this study. Otherwise, the elevation of FGF-23 may suppress PTH secretion [26] in the early stage of CKD; it resulted in no increase of iPTH levels in our animals, which exhibited declined kidney function. The results of serum iPTH and FGF-23 levels were also compatible with the fact that the serum FGF-23 levels change before the serum iPTH levels when the kidney function is deteriorating [27]. With regards to the serum levels of alkaline phosphatase (ALP), an osteoblast activity marker, no significant difference was seen among the three groups (Figure 1C). The serum sclerostin levels were significantly higher in rats from the CKD group (309 ± 38.1 pg/mL) than those in rats from the CKD + exercise group (157.1 ± 21.1 pg/mL) (Figure 1D). The serum sclerostin data in the present study indicated that exercise had an influence on sclerostin production during conditions of CKD. The serum levels of collagen type I C-telopeptide (CTX-1), a bone resorption marker, showed no significant difference when the data for the CKD group (156.5 ± 12.0 pg/mL) were compared with those for the two other groups (CKD + exercise, 111.4 ± 11.5 pg/mL; sham, 109.72 ± 12.3 pg/mL). The high serum CTX-1 levels in the rats from the CKD group indicated that the CKD rats had higher bone resorption activity (Figure 1E). Lower CTX-1 levels in the rats from the CKD + exercise group relative to those from the CKD group indicated that exercise alleviated bone resorption in the rats with declined kidney function.

### 2.3. Rats from the CKD + Exercise Group Had a Better Bone Mineral Density (BMD) and Bone Volume Parameters than Those from the CKD Group

**Figure 2:**
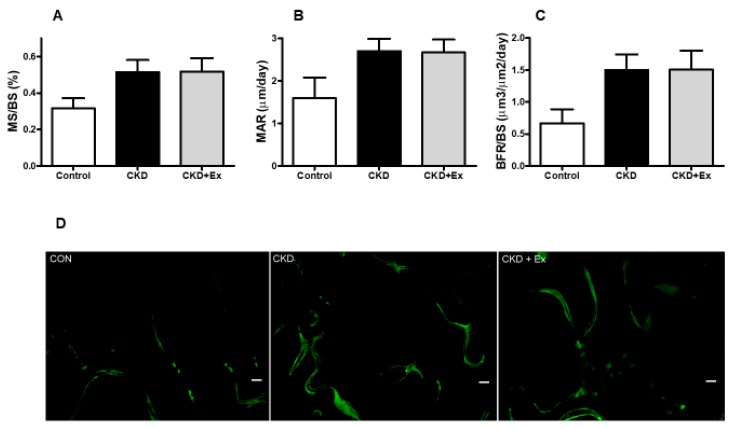
Dynamic bone histomorphometric parameters and micro-CT bone scanning. Dynamic bone histomorphometric parameters (**A**–**C**), and dynamic histology (**D**) did not show significant differences between the CKD and CKD + exercise groups. *N* = 8, 13, and 9 in the control, CKD, and CKD + exercise groups, respectively. Mineralization over bone surface, MS/BS; Mineral apposition rate, MAR; Bone formation rate per bone surface, BFR/BS. All data are represented as mean ± SEM. Scale bar = 50 μm.

**Figure 3:**
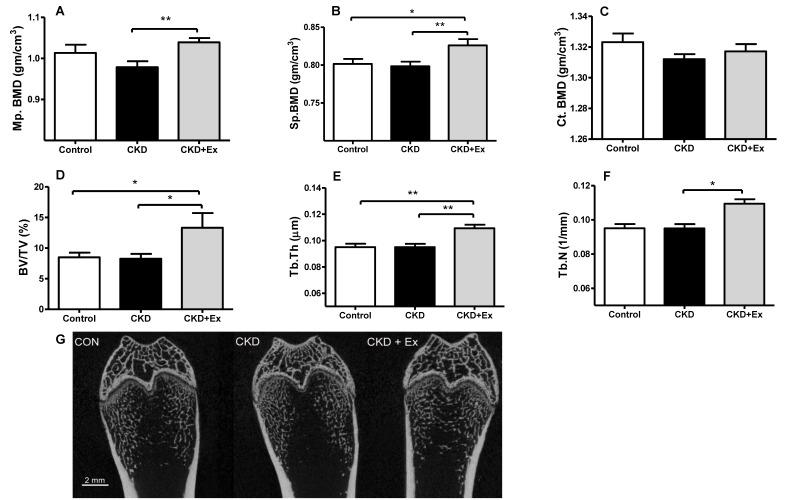
Bone mineral density (BMD) and bone microarchitecture parameters. Distal femoral metaphysis (Mp.) and L5 vertebrate (Sp.) bone mineral density was significantly higher in rats from the CKD + exercise group than in rats from the CKD group (**A**,**B**). Cortical bone density did not show significant differences between the groups (**C**). Trabecular bone volume (BV/TV), trabecular thickness (Tb.Th), and trabecular number (Tb.N) were significantly higher in rats from the CKD + exercise group (**D**–**F**), relative to those from the CKD group. Micro-CT scanning demonstrated that the trabecular bone in rats from the CKD group was looser than that in rats from the CKD + exercise group (**G**). *N* = 8, 13, and 9 in the control, CKD, and CKD + exercise groups, respectively. Values are represented as mean ± SEM. * *p* < 0.05, ** *p* < 0.01. Scale bar = 2 mm.

In the dynamic bone histomorphometric analysis, mineralization over bone surface (MS/BS), mineral apposition rate (MAR), and bone formation rate per bone surface (BFR/BS) showed no significant differences between the CKD and CKD + exercise groups (Figure 2A–C). Rats that underwent partial nephrectomy showed an increasing trend of bone formation rate compared with rats from the control group [28]. In the micro computed tomography (micro-CT) scanning analysis of the bones, the CKD + exercise-group rats had a higher BMD in the distal femoral metaphysis (1.038 ± 0.01 vs 0.978 ± 0.146 gm/cm^3^) and L5 vertebrae (0.826 ± 0.008 vs 0.798 ± 0.006 gm/cm^3^), compared to the rats in the CKD group (Figure 3A,B). Cortical BMD did not show significant differences (Figure 3C). This may be associated with the fact that in our experiment, the time to distinguish the differences in the lower bone remodeling region was limited. In the trabecular bone microarchitecture analysis of the distal femur, the following parameters were measured: bone volume ratio (BV/TV), trabecular thickness (Tb.Th), trabecular number (Tb.N), and trabecular separation (Tb.Sp) (space between trabeculae). The values of the trabecular bone microarchitecture parameters in rats from the CKD + exercise group were higher when compared to the rats from the CKD groups (Figure 3D,E,F). The Tb.Sp did not show a significant difference. These data indicated that the rats from the CKD + exercise group had a better bone microarchitecture than the rats from the CKD group.

### 2.4. Immunohistochemistry

**Figure 4:**
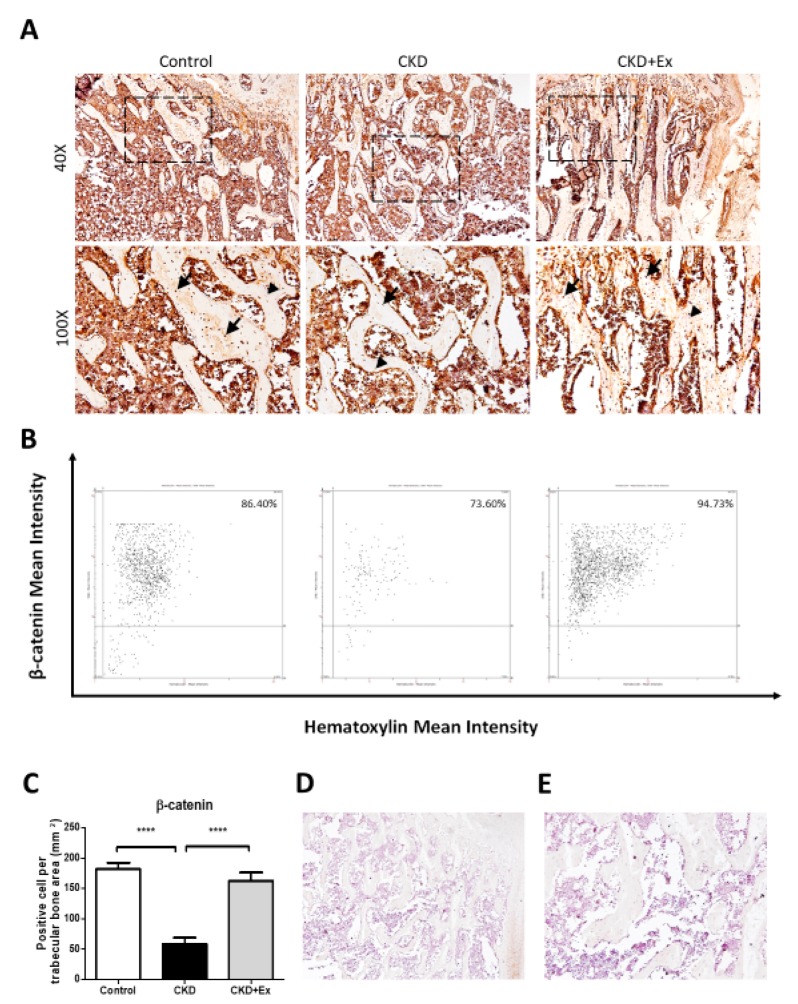
β-catenin immunohistochemistry on osteocytes in the tibial trabecular bone. (**A**) Protein expression of β-catenin in the trabecular bone of rats was determined by IHC assay. Rats from the CKD + exercise group showed a higher β-catenin staining signal on osteocytes than in case of rats from the CKD group. The arrows indicate the positive cells. The arrowheads indicate the negative cells. (**B**) Representative images of tissue cytometry using HistoQuest software. β-catenin-immunoreactive (IR) positive and negative cells were counted and the signal intensity was quantified. (**C**) Images are representative and quantification data are presented as the mean ± SD from 5 rats in each group. The CKD group showed a significant difference compared to the CKD + exercise and sham groups (**** *p* < 0.0001). The CKD + exercise group showed no significant difference compared to the sham group. The analysis was performed using the One way-ANOVA test. (**D**) 40× and (**E**) 100× images of the negative control without primary antibody.

Immunohistochemistry analysis demonstrated that the β-catenin signal was higher in rats from the CKD + exercise group than in rats from the CKD group (Figure 4). The results indicated that the Wnt/β-catenin signaling pathway was activated after exercise training in the rats with declined kidney function.

## 3. Discussion

In our study, we established a rat model of mild CKD and divided these rats into two groups: the CKD group and the CKD + exercise group. In the rats from both these groups, the serum FGF-23 levels increased, compared to those from the sham group. Serum iPTH, phosphate, and calcium levels in the rats with kidney dysfunction did not change relative to the rats from the sham group. Increased FEP in the rats with declined kidney function was observed. The above data were compatible with the appearance of mild CKD. After exercise training, rats from the CKD + exercise group had a lesser osteoporosis appearance than those from the CKD group. The serum sclerostin levels decreased after exercise intervention, i.e., in the rats subjected to exercise, when compared with those without exercise.

Exercise has been reported to improve osteoporosis in aged and post-menopausal women [24]. However, CKD patients, who show chronic metabolic dysfunction, represent a specific population, and should not be extrapolated to populations from other studies. In clinical practice, physicians have raised a concern that exercise may aggravate the burden in CKD patients, and are often conservative to related studies. However, Kidney Disease: Improving Global Outcomes (KDIGO) Clinical Practice Guidelines for CKD suggest that compatible physical activity should be undertaken in CKD individuals [29]. Several studies about exercise in pre-dialysis CKD patients were conducted and it was demonstrated that at least in mild CKD patients, exercise is safe [30,31]. Exercise is considered beneficial to vascular health in a CKD-affected population [32,33]. However, studies about the effects of exercise on CKD–MBD are limited. We established a mild CKD rat model to evaluate the influence of moderate-intensity treadmill exercise [34] on CKD–MBD. In our study, the mild CKD-affected rats showed improvements with regards to BMD and skeletal microarchitecture following exercise intervention. Serum concentrations of the bone resorption marker CTX-1 [35] were higher in rats from the CKD group, and showed an improvement in rats from the CKD + exercise group, indicating that exercise alleviated bone resorption in the rats with mild CKD. However, exercise did not alter the dynamic histomorphometric parameters and serum ALP levels, when the rats from the CKD group were compared to the rats from the CKD + exercise group. Collectively, moderate-intensity exercise lessened bone resorption, but did not alter the bone formation rate in our mild CKD animal model.

Bone, a mineral repository, helps maintain the serum calcium and phosphate homeostasis [7]. When the kidney function deteriorates, the bone–parathyroid–kidney axis is disrupted, and subsequently, various mineral and hormonal disorders occur. PTH suppresses the inhibitory effects of sclerostin on osteocytes [36,37]. Increased sclerostin levels in uremic patients may cause PTH resistance and further increase PTH production [38]. Decreased sclerostin levels may help control mineral or hormonal disorders [39]. Early in CKD, sclerostin plays a more important role in bone resorption than the parathyroid gland [40]. Our study on a mild CKD model also demonstrated that the serum levels of sclerostin, and not iPTH, changed in the rats from the CKD group. Exercise training for the rats with declined kidney function decreased the serum sclerostin levels and bone resorption, but did not alter the serum FGF-23, iPTH, phosphate, and calcium levels. FEP, a phosphate retention indicator, was also not different between the rats in the CKD + exercise and CKD groups. According to the above data, exercise intervention did not improve the mineral disturbance, abnormal FGF-23 elevation, and serum PTH concentration in the mild CKD model within our experimental period. However, in the present study, FECa was lower in the rats from the CKD + exercise group than in the rats from the CKD group (without exercise). Decrease in bone resorption may contribute to the decreased urinary excretion of calcium. Moreover, decreased FECa in sost−/− knockout mice has been reported [39]. Lower sclerostin levels in the CKD rats that underwent exercise training may also help reduce calcium excretion from the kidneys. Increased excretion of urinary calcium that developed into secondary hyperparathyroidism has also been reported [41]. Loss of calcium from urine, triggering the elevation of the parathyroid hormone levels, was considered. The probable effect of exercise, whereby it inhibits the further increase of the parathyroid hormone levels by decreasing the production of sclerostin or calcium loss, may need to be confirmed by performing studies with a longer observation time. 

The Wnt/β-catenin signal pathway has an important role in bone formation and bone resorption. Osteocytes regulate the canonical Wnt/β-catenin signal pathway to promote osteoblastogenesis and suppress osteoclastogenesis [13]. Combined with the above effects, the Wnt/β-catenin signal pathway contributes to bone mass increase. Sclerostin, which is secreted by osteocytes, inhibits the Wnt/β-catenin signaling pathway in bones by binding to the LRP5/6 receptor [42]. Due to the blockade by sclerostin, Wnt is unable to bind to the frizzled protein-LRP5/6 receptor complex and activate downstream β-catenin signaling. Serum sclerostin levels rise when the kidney function declines. Elevated serum sclerostin levels and decreased Wnt/β-catenin signaling occur in the early stage of CKD [10]. Sclerostin correlates with BMD and bone metabolism markers in hemodialysis patients [43] and participates in osteoporosis during early CKD [40]. Mechanical stimulation reduces the expression of sclerostin by osteocytes [22]. Physical activity is important and affects the serum sclerostin levels and bone mineral content [25,44,45]. In our mild CKD rodent model, exercise training alleviated the rise in serum sclerostin levels. However, the bone formation rate did not differ. Instead, bone resorption improved after exercise intervention. In a study regarding the evaluation of the protective effects of Wnt/β-catenin signaling against glucocorticoid-induced osteoporosis, the activation of Wnt/β-catenin signaling in sclerostin knockout mice prevented bone resorption instead of the restoration of bone formation [46]. This result was compatible with our findings in the present study. Probably under hypercatabolic conditions, Wnt/β-catenin signaling modulates the catabolic pathway prior to the anabolic pathway in bones.

In our study, we established a mild CKD model and demonstrated that moderate intensity exercise was beneficial to bone health. However, one limitation of our study is that whether the data in this study could be extrapolated to moderate and severe CKD cases is not yet clear. Although CKD patients are encouraged to perform endurance exercise [29,33], the compensating ability for burden developed from exercise in pre-dialysis patients should be assessed with caution; further studies on pre-dialysis patients are necessary. However, our study indicated that exercise training could modulate osteocytes and regulate bone resorption in CKD. Modulating the Wnt/β-catenin signaling pathway provides an alternative therapeutic strategy for bone disorders in CKD. However, anti-sclerostin antibodies [47], which stimulate vascular calcification in CKD patients after their application, raises concerns [38,48]. For this reason, performing exercises that have been proven to be advantageous to blood vessels [49] is a safe way to decrease the serum sclerostin levels and improve bone mass in mild CKD.

## 4. Materials and Methods

### 4.1. Animals

Male Sprague Dawley rats were obtained and housed under controlled conditions (room temperature, 22 ± 1 °C; alternating 12-h light and dark periods). Before starting the experiment, all animals were acclimatized to the environment for one week. The present experimental animals were fed rat chow diet (Purina Rodent Chow 5001, Labdiet, Richmond, IN, USA), containing 0.95% calcium and 1.07% phosphate, and tap water ad libitum throughout the study. This study was approved by the University Institutional Animal Care and Use Committee of National Cheng Kung University (NCKU) (Project code—105108, date of approval—23 Dec. 2016). The care and handling of the animals were in accordance with the National Institute of Health guidelines for the ethical treatment of animals. The surgical procedures were performed under anesthesia, and all efforts were made to minimize suffering. 

### 4.2. Mild CKD Animal Model with Exercise Training

At the age of eight weeks, the rats were randomly divided into the CKD and sham groups. A two-step procedure of partial nephrectomy was performed to induce CKD. At the first week, we excised one-third of the right kidneys of the rats. In the subsequent week, the entire left kidney was removed from the rats. After partial nephrectomy, the CKD rats were randomized into the exercise and non-exercise groups. The exercise program was performed by making the rats run on motorized treadmills (TD.06653; Harlan Teklad, Madison, WI, USA) for eight weeks. In the exercise program for the present study, the rats were made to run on the treadmills for 5 days per week. At the first week of the exercise program, the rats were trained by being made to run at the rate of 9 m/min for 10 min per day. At the second week of training, the rats ran at the rate of 12 m/min for 1 h on each exercise day. After being acclimatized to the running program, the rats in the exercise group ran at a rate of 16 m/min for 1 h for the remaining weeks of the running program. 

### 4.3. Serum and Urine Assays

At the time of animal sacrifice, the serum and spot urine were collected and stored at −80 °C immediately until further analysis. Serum blood urea nitrogen (BUN), creatinine, phosphate, calcium, and ALP levels were analyzed by an automatic chemistry analyzer. Enzyme-linked immunosorbent assay kits were used to measure the levels of serum iPTH (Immutopics, Inc., San Clemente, CA, USA), FGF-23 (Kainos Laboratories, Tokyo, Japan), and C-telopeptide (CTX-1) (MyBioSource, Inc., San Diego, CA, USA). Urine chemistry values were measured by an automatic chemistry analyzer. Urinary FEP and FECa were calculated [28].

### 4.4. Micro Computed Tomography (Micro-CT) and Dynamic Bone Histomorphometry Analysis

The rats were injected intraperitoneally with calcein at a dose of 20 mg/kg (Sigma-Aldrich, St Louis, MO, USA) 10 days and 3 days before being euthanized. After death, the right femurs and lumbar vertebrae (L5) were dissected and fixed in 70% ethanol. The bone samples were subjected to 3D scanning using a micro-CT scanner (Skyscan 1176, SKYSCAN, Kontich, Belgium) using the following conditions: 0.5 mm aluminum filter, 48 kev, 200 µA, and 1°/picture with a 2600-minisecond exposure time and pixel size of 8.973 µm. Cross-sectional images (8-bit BMP file) of each sample were reconstructed using NRecon software (version 1.6.9.8; Skyscan); the software was set up using the following parameters: dynamic range = 0–0.06, smoothing = 2, ring artifact correction = 20, beam hardening correction (%) = 40. Densitometric and histomorphometric analyses were performed using a CT-Analyzer (version 1.12.0.0; Skyscan) with a consistent gray threshold range (80–255) selected for all sample images to obtain the BMD and bone microarchitecture parameters. Volumetric BMD (vBMD, g/cm^3^) was measured on the metaphysis area, spongy area, and cortical bone (area selected from transverse slices 1 mm in thickness at the midshaft femur). The metaphysis region was defined as the region located 1–4 mm below the growth plate of the distal femur. The trabecular bone region of the right distal femoral metaphysis was chosen for the analysis of the microarchitectural parameters. Cortical bone measurement was conducted on the middle of the diaphysis. Bone microarchitecture analysis was performed to measure the bone volume ratio (BV/TV) (%), trabecular thickness (Tb.Th) (μm), trabecular number (Tb.N) (1/mm), and trabecular separation (Tb.Sp) (space between trabeculae, μm). After micro-CT scanning, the femoral bone samples were embedded in methylmethacrylate (MMA). Frontal cutting (5 μm) of the MMA-embedded proximal tibiae was performed using an automatic rotary microtome (HM 355S; Thermo Scientific, Waltham, MA, USA). Metaphyseal cancellous bone of the proximal tibiae (1–4 mm below the growth plate) were photographed under a fluorescent microscope at 100× magnification. Length and thickness measurements of the fluorescently labeled/non-labeled bone surfaces were performed with the obtained images using Image Pro Plus (version 6.1; Media Cybernetics, Rockville, MD, USA). Three dynamic histomorphometric parameters—bone mineralization over bone surface (MS/BS, %), mineral apposition rate (MAR, μm/day), and bone formation rate per bone surface (BFR/BS, μm^3^/μm^2^/day)—were measured in the periosteal surfaces and endocortical surfaces, as described in a previous study [28,50]. 

### 4.5. Immunohistochemistry

The right tibial bone isolated from each rat was fixed in a 3.7% neutral paraformaldehyde solution for 24 h. Then, all fixed samples were decalcified with a 10% EDTA solution (pH 7.4) at 4 °C for 35–40 days. Once decalcified, each bone was paraffin embedded, and sectioned for the following histologic analyses. After being deparaffinized by Xylene, rehydrated in graded alcohol solutions, and antigen retrieved with 0.1% trypsin, the sections were immersed in 0.3% H_2_O_2_ in order to inhibit endogenous peroxidase activity for 30 minutes, and subsequently blocked using a solution composed of 5% NS + 0.5% triton X-100 for 1 h at room temperature. Then, the tissues were incubated overnight at 4 °C with polyclonal β-catenin antibodies (Sigma-Aldrich, St Louis, MO, USA) at a 1:100 dilution. The slides were subsequently incubated with the corresponding biotinylated secondary antibodies (HRP anti-rabbit for β-catenin) at a 1:300 dilution for 1 h at room temperature, followed by visualization using diaminobenzidine (DAKO). Hematoxylin staining (2 min) was then conducted, and the slides were mounted using Entellan mounting medium + xylene solution (1:1). Negative controls were established for each bone section by omitting the primary antibody treatment. To quantify the bone expression of β-catenin in the osteocytes of the trabecular bone, the osteocytes stained were classified as positive or negative cells, and the total number of trabecular osteocytes with positive staining was counted and normalized with the bone area. The quantification was conducted by counting 10 random areas in the tibial trabecular bone and using samples from five rats in each group. The number of immunoreactive cells labeled for β-catenin in the automatic optical inspection (AOI) were calculated. The amounts of active β-catenin-immunoreactive cells were expressed as percentages of immunoreactive cells in all cells. The staining intensity was quantified by tissue cytometry using HistoQuest analysis software (TissueGnostics, Tarzana, CA, USA). Antibody-mediated chromogen stain and the counter-stain were separated by HistoQuest software and displayed in scattergrams [51].

### 4.6. Statistical Analysis

All statistical tests were performed using statistical software (SPSS, version 17.0, IBM Corporation, Armonk, NY, USA). Results were represented as the mean ± SEM. One way-ANOVA and LSD post hoc method tests were used to determine statistically significant differences between the groups. Two-sided *p* values < 0.05 were considered statistically significant. 

## 5. Conclusions

In the current mild CKD animal model, moderate-intensity exercise reduced serum sclerostin levels and bone resorption rate; BMD and cancellous bone volume parameters were also ameliorated following exercise. Thus, exercise training could serve as an alternative therapeutic strategy for treating osteoporosis in CKD patients.

## Figures and Tables

**Table 1:** Serum and urinary biochemistries of the experimental animals at the time of sacrifice.

	Groups	Control (Sham) *n* = 8	CKD *n* = 13	CKD + Exercise *n* = 9
Measurements				
Body weight at sacrifice (g)		569.0 ± 17.4	515.69 ± 16.56a	511.28 ± 16.85a
BUN (mg/dL)		21.56 ± 0.85	40.78 ± 3.01b	40.38 ± 3.92b
Creatinine (mg/dL)		0.53 ± 0.02	0.81 ± 0.037b	0.73 ± 0.036b
Phosphate (mg/dL)		6.59 ± 0.43	7.04 ± 0.34	6.56 ± 0.26
Calcium (mg/dL)		9.21 ± 0.12	9.72 ± 0.21	9.39 ± 0.13
Urine FEP (%)		1.89 ± 0.32	7.43 ± 1.74a	9.32 ± 2.08b
Urine FECa (%)		0.89 ± 0.1c	2.11 ± 0.4	0.66 ± 0.11c

Values represented as mean ± SEM; available urine was collected from the rats in the control (*n* = 6), the CKD (*n* = 4), and the CKD + exercise groups (*n* = 7). a, Compared to control group, *p* < 0.05; b, Compared to control group, *p* < 0.01; c, Compared to CKD group, *p* < 0.01. CKD is chronic kidney disease; BUN is blood urea nitrogen; FEP is fractional excretion of calcium.

## Abbreviations

CKD	Chronic kidney disease
CKD–MBD	Chronic kidney disease–mineral bone disorder
BUN	Blood urea nitrogen
FEP	Fractional excretion of phosphate
FECa	Fractional excretion of calcium
FGF-23	Fibroblast growth factor-23
iPTH	Intact parathyroid hormone
ALP	Alkaline-phosphatase
CTX-1	Collagen type I C-telopeptide
MS/BS	Mineralization over bone surface
MAR	Mineral apposition rate
BFR/BS	Bone formation rate per bone surface
Micro-CT	Micro computed tomography
BMD	Bone mineral density
BV/TV	Bone volume ratio
Tb.Th	Trabecular thickness
Tb.N	Trabecular number
Tb.Sp	Trabecular separation
KDIGO	Kidney Disease: Improving Global Outcomes
LRP5/6	Low-density lipoprotein receptor-related protein 5/6
